# Óbitos Intra e Extra-Hospitalares por Infarto Agudo do Miocárdio nas Capitais Brasileiras

**DOI:** 10.36660/abc.20200043

**Published:** 2021-08-09

**Authors:** Sterffeson Lamare Lucena de Abreu, Joana D’Arc Matos França de Abreu, Maria dos Remédios Freitas Carvalho Branco, Alcione Miranda dos Santos

**Affiliations:** 1 Universidade Federal do Maranhão Hospital Universitário São LuísMA Brasil Hospital Universitário da Universidade Federal do Maranhão, São Luís, MA - Brasil; 2 Universidade Federal do Maranhão Pós Graduação em Saúde Coletiva São LuísMA Brasil Universidade Federal do Maranhão - Pós Graduação em Saúde Coletiva, São Luís, MA - Brasil

**Keywords:** Infarto do Miocárdio, Extra-Hospitalar, Epidemiologa, Óbitos, Indicadores Demográficos, Indicadores Sociais, Mortalidade, Morte Súbita

## Abstract

**Fundamento:**

O infarto agudo do miocárdio (IAM) é a principal causa de óbito no Brasil e no mundo. Aproximadamente metade dos óbitos ocorrem fora do ambiente hospitalar.

**Objetivos:**

Analisar a distribuição, a evolução temporal e as características sociodemográficas (CSD) dos óbitos intra e extra-hospitalares por IAM nas capitais brasileiras e a sua relação com indicadores municipais de desenvolvimento (IMD).

**Métodos:**

Estudo ecológico com contagem anual dos óbitos por IAM nas 27 capitais brasileiras de 2007 a 2016, os quais foram divididos em dois grupos, intra-hospitalar (H) e extra-hospitalar (EH). Avaliou-se a evolução temporal das taxas de mortalidade em cada grupo e as diferenças das CSD. Modelos de regressão binominal negativa compararam temporalmente a contagem de óbitos em cada grupo com as seguintes variáveis: residir nas regiões Sul e Sudeste (S/SE), índice de desenvolvimento humano municipal (IDHM), índice de Gini e expectativa de anos de estudo (EAE). Considerou-se estatisticamente valores significativos de p < 0,05.

**Resultados:**

A taxa de mortalidade EH para o conjunto das capitais aumentou ao longo do tempo. Todas as CSD pesquisadas foram difententes entre os grupos (p < 0,001). No grupo EH prevaleceram os óbitos em homens, em pacientes ≥ 80 anos e em solteiros. O S/SE elevou a incidência de óbitos extra-hospitalares (IRR = 2,84; IC 95% = 1,67-4,85), enquanto o maior EAE registrou queda (IRR = 0,86; IC 95% = 0,77-0,97). Para o grupo H, o maior IDHM reduziu a incidência de óbitos (IRR = 0,44; IC 95% = 0,33-0,58), enquanto o maior EAE apresentou crescimento (IRR = 1,09; IC 95% = 1,03-1,15).

**Conclusão:**

Os óbitos intra e extra-hospitalares por IAM nas capitais apresentam diferenças sociodemográficas, incidência influenciada por IMD e progressivo aumento da ocorrência extra-hospitalar.

## Introdução

O infarto agudo do miocárdio (IAM) é a principal causa individual de óbito no Brasil e no mundo^1^^,^^2^ com taxas de mortalidade média de 30% quando não há tratamento e menor que 6% com o emprego da terapia apropriada em tempo hábil.^3^ Metade destes óbitos ocorrem em até duas horas do início do quadro e 80% nas primeiras 24 horas, tendo como consequência um grande número de óbitos antes de qualquer antendimento hospitalar.^4^

O tratamento específico e adequado para IAM é de alto custo e sua disponibilidade concentra-se nos maiores centros, principalmente nas capitais, realidade esta mais evidente nas regiões Norte, Nordeste e Centro-Oeste do Brasil.^5^ Ainda que estudos epidemiológicos mostrem que a mortalidade por IAM está lentamente reduzindo no mundo, esta queda é lenta em países com menor Produto Interno Bruto (PIB), entre as classes sociais mais pobres e em bairros com menores menores condições socioeconômicas.^6^^–^^8^

Existem poucos estudos publicados sobre os óbitos extra-hospitalares por IAM. A maioria dos trabalhos aborda a mortalidade geral, sem distingui-la entre intra e/ou extra-hospitalar. As pesquisas clínicas sobre os fatores de risco são realizadas com os pacientes que receberam tratamento hospitalar. Ainda é desconhecido se os óbitos externos apresentam diferenças sociodemográficas em relação aos que ocorrem no ambiente hospitalar, bem como ainda não está bem explicado se os fatores locais e ambientais apresentam associação à mortalidade extra-hospitalar.^9^^,^^10^

O objetivo deste estudo é analisar temporalmente os óbitos intra e extra-hospitalares por IAM nas capitais brasileiras, identificando as diferenças sociodemográficas e as que estão relacionadas aos índices municipais de desenvolvimento. Escolhemos analisar apenas as capitais porque todas dispõem atualmente de tratamento avançado para o IAM.^11^

## Método

Realizou-se um estudo ecológico dos casos de óbito por IAM ocorridos nas 27 capitais brasileiras no período de 2007 a 2016. Os dados dos óbitos por capital (local de ocorrência intra ou extra hospitalar, sexo, faixa etária, escolaridade, estado civil e cor da pele) foram obtidos mediante consulta ao Sistema de Informação sobre Mortalidade (SIM), plataforma *on-line* criada pelo Departamento de Informática do SUS (Datasus) para a obtenção regular de dados sobre a mortalidade no Brasil. Os óbitos foram divididos em dois grupos e de acordo com o local de ocorrência: intra-hospitalar ou extra-hospitalar.

Para a seleção dos óbitos por IAM no SIM, foram considerados os registros que tiveram como causa básica IAM (CID 10: I 21). Os óbitos com local de ocorrência ignorado não foram incluídos no estudo. As taxas de mortalidade intra-hospitalar e extra-hospitalar foram obtidas pela razão do número de óbitos por IAM e a população de cada capital brasileira (a cada 100.000 habitantes). As referidas taxas são apresentadas por média, desvio-padrão (DP) e valores mínimo e máximo.

Para avaliar a evolução temporal da taxa de mortalidade nos dois grupos, foram calculadas as taxas anuais de mortalidade intra e extra-hospitalar no conjunto de todas as capitais brasileiras. A população foi corrigida por interpolação e extrapolação linear entre os dados dos censos demográficos de 2000, 2010 e da projeção da população em 2017, disponibilizados pelo Instituto Brasileiro de Geografia e Estatística (IBGE). As taxas são apresentadas por óbitos a cada 100.000 habitantes e expressas em gráfico de linhas.

A plataforma Atlas Brasil, do Programa das Nações Unidas para o Desenvolvimento (PNUD), foi utilizada para obter as variáveis independentes (IDHM, índice de Gini e expectativa de anos de estudo), além de informar o tamanho da população de cada capital.^12^

### Análise estatística

Para comparar o número de óbitos nos dois grupos, segundo as características sociodemográficas (sexo, faixa etária, escolaridade, estado civil e cor da pele), foi utilizado o teste qui-quadrado. As características sociodemográficas foram apresentadas através de frequências absolutas e relativas. Para mostrar o impacto de cada característica, foi feita a análise dos resíduos padronizados do teste qui-quadrado, os quais estão expressos como Z na Tabela 2. Considerando um nível de significância de 5%, os valores de Z > +1,96 ou < −1,96 são estatísticamente significativos e o sinais positivo e negativo mostram as diferenças entre os grupos.

Para verificar quais as variáveis independentes estavam associadas ao número de óbitos nos dois grupos, foi utilizada a metodologia de dados em painel, na qual a informação de várias unidades amostrais (cada capital) são analisadas ao longo do tempo, ou seja, as observações são consideradas em duas dimensões: a unidade amostral e o tempo.^13^ Desta forma, foram ajustados os modelos de regressão de Poisson e binomial negativa. Os ajustes temporal e ponderado para cada um dos grupos foram feitos pelo tamanho da população de cada capital, cuja ponderação foi utilizada para que cada unidade de amostra tivesse o mesmo peso na avaliação das associações.

Os modelos foram testados com efeitos fixos e aleatórios. Os modelos de primeiro tipo possibilitam que cada capital tenha o seu prórprio intercepto, que serve como controle e permite o ajuste para variáveis não mensuradas e que não mudam ao longo do tempo. É o caso dos dados censitários, que são atualizados apenas a cada dez anos.^13^

Para a escolha do modelo que melhor se ajusta aos dados foi considerado o critério de informação de Akaike (AIC);^14^ quanto menor o AIC, melhor o ajuste. Também foram estimados a razão de incidência (IRR) e seu respectivo intervalo de confiança, considerando como referência do intervalo de confiança o valor percentual de 95% (IC 95%). A análise estatística foi feita através do *software* Stata^®^ 14.0.

Esta pesquisa utilizou apenas dados de domínio público irrestrito, prescindindo de apreciação por Comitê de Ética e Pesquisa por não se enquadrar nos termos da Resolução nº 466, de 12 de dezembro de 2012.^15^

## Resultados

**Figura 1:**
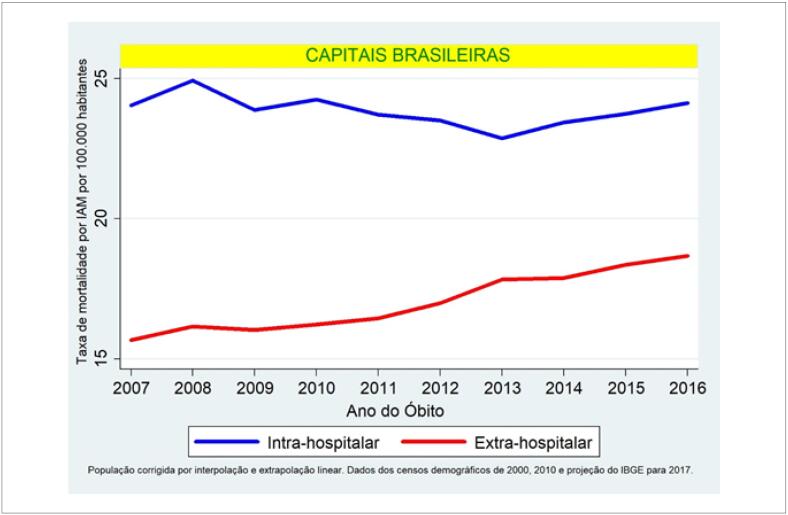
Evolução temporal das taxas de mortalidade intra e extra-hospitalar por infarto agudo do miocáridio por 100.000 habitantes. Capitais brasileiras, 2007-2016.

Ocorreram 189.634 óbitos por IAM nas capitais brasileiras de 2007 a 2016, 41,7% destes foram de caráter extra-hospitalar. A taxa média de mortalidade a cada 100.000 habitantes nas capitais brasileiras foi de 25,2 ± 1,3 para o intra-hospitalar e de 18 ± 1,2 para o extra-hospitalar. A evolução temporal da taxa anual para o conjunto de todas as capitais em ambos os grupos está demonstrada na Figura 1.

Fonte: Datasus. Estatísticas vitais.

As maiores e menores taxas médias de óbitos foram registradas, respectivamente, em Recife (43,2%) e em Palmas (8,7%) para o grupo intra-hospitalar, e no Rio de Janeiro (33,8%) e em Macapá (4,7%) para o grupo extra-hospitalar (Tabela 1). Em várias capitais os óbitos extra-hospitalares foram mais prevalentes que os intra-hospitalares: Palmas, São Luís, Rio de Janeiro, Curitiba, Florianópolis, Porto Alegre e Campo Grande.

**Tabela 1:** Taxas de mortalidade por infarto agudo do miocárdio nas capitais brasileiras de 2007 a 2016 (óbitos/100.000 habitantes). Média, desvio padrão (DP) e valores mínimos e máximos registrados

	Intra-hospitalar (H)			Extra-hospitalar (EH)			% EH
	Média	DP	Min-Max	Média	DP	Min–Max	Média
Porto Velho	13,12	2,38	10,15 – 18,53	12,14	5,34	6,28 – 22,40	48,06%
Rio Branco	14,17	3,40	10,42 – 19,33	10,04	3,52	5,95 – 14,88	41,47%
Manaus	14,35	1,86	11,71 – 17,32	4,88	2,07	2,22 – 9,10	25,38%
Boa Vista	12,49	2,83	7,39 – 16,18	9,35	1,71	5,63 – 11,61	42,81%
Belém	18,51	3,07	14,99 – 23,47	17,53	3,67	12,49 – 22,39	48,64%
Macapá	10,44	3,26	5,52 – 16,83	4,74	3,56	1,00 – 11,55	31,23%
Palmas	8,68	2,26	4,82 – 11,65	13,43	5,11	5,69 – 22,57	60,74%
São Luís	18,66	2,15	15,96 – 21,93	20,17	4,45	13,30 – 26,90	51,94%
Teresina	21,40	1,85	18,08 – 25,05	13,84	2,96	10,93 – 20,90	39,27%
Fortaleza	16,88	1,33	14,35 – 18,19	6,38	1,86	3,71 – 9,42	27,43%
Natal	23,46	2,68	21,52 – 30,11	23,40	5,75	16,32 – 31,85	49,94%
João Pessoa	25,17	1,70	21,84 – 27,37	21,76	3,01	17,97 – 25,98	46,37%
Recife	43,16	5,54	36,96 – 51,37	21,23	2,21	15,95 – 23,61	32,97%
Maceió	17,77	1,91	14,69 – 20,05	14,20	2,94	10,29 – 18,80	44,42%
Aracajú	17,02	1,82	14,53 – 20,37	11,82	2,80	8,58 – 18,38	40,98%
Salvador	16,19	1,48	13,04 – 17,98	9,47	1,72	6,65 – 13,49	36,91%
Belo Horizonte	15,00	1,48	13,01 – 17,56	9,11	0,60	7,75 – 9,94	37,79%
Vitória	21,70	4,37	15,56 – 27,65	18,34	1,40	16,04 – 19,83	45,80%
Rio de Janeiro	32,68	2,55	29,35 – 38,17	33,75	2,61	27,93 – 36,72	50,81%
São Paulo	36,41	2,07	33,62 – 39,72	17,84	1,73	15,84 – 20,66	32,88%
Curitiba	16,87	1,71	14,56 – 18,84	23,42	1,84	20,49 – 25,86	58,13%
Florianópolis	16,55	2,34	12,58 – 19,84	16,95	4,00	10,92 – 24,22	50,60%
Porto Alegre	23,22	1,65	21,07 – 26,90	30,46	3,18	25,33 – 34,84	56,74%
Campo Grande	18,00	1,70	16,12 – 21,48	33,30	10,59	22,75 – 56,81	64,91%
Cuiabá	20,42	1,78	18,62 – 24,13	15,49	4,72	10,52 – 23,59	43,14%
Goiânia	17,57	2,55	13,44 – 21,89	13,43	3,21	9,52 – 19,34	43,32%
Brasília	15,86	1,19	14,36 – 18,23	7,55	2,46	4,20 – 12,10	32,25%

Fonte: Datasus. Estatísticas vitais.

Os dois grupos mostraram-se estatisticamente diferentes em todas as características sociodemográficas pesquisadas (Tabela 2). Comparativamente, houve mais óbitos do sexo masculino no grupo extra-hospitalar (57,4% a 55,5%). Quanto à faixa etária no grupo extra-hospitalar, predominou os > 80 anos (29,7% a 26,3%). A morte de pacientes casados se mostrou menor fora do ambiente hospitalar (38% a 46%), como mostra a Tabela 2.

**Tabela 2:** Distribuição sociodemográfica dos óbitos intra e extra-hospitalares por infarto agudo do miocárdio. Capitais brasileiras, 2007-2016

	Intra-hospitalar			Extra-hospitalar			Valor de p*
	N (110.549)	%	Z†	N (79.085)	%	Z†	
**Sexo**							< 0,001
Masculino	61.304	55,45	–3,58	45.389	57,39	4,24	
Feminino	49.245	44,55	4,06	33.696	42,61	–4,81	
**Faixa etária**							< 0,001
< 1 ano	50	0,05	3,43	3	0	–4,06	
1 – 4 anos	3	0	0,95	0	0	–1,12	
5 – 9 anos	2	0	0,19	1	0	–0,22	
10 – 14 anos	14	0,01	–0,15	11	0,01	0,18	
15 – 19 anos	207	0,19	3,73	67	0,08	–4,42	
20 – 29 anos	685	0,62	0,97	447	0,57	–1,14	
30 – 39 anos	1.877	1,7	–6,02	1.821	2,31	7,12	
40 – 49 anos	6.991	6,33	–6,10	5.904	7,47	7,22	
50 – 59 anos	17.580	15,91	–0,98	12.788	16,19	1,16	
60 – 69 anos	25.204	22,81	4,73	16.745	21,20	–5,60	
70 – 79 anos	28.847	26,10	10,22	17.729	22,45	–12,09	
≥ 80 anos	29.052	26,29	–9,02	23.471	29,72	10,67	
**Estado civil**							< 0,001
Solteiro	20.517	19,73	–17,49	19.489	25,82	20,53	
Casado	47.417	45,60	15,72	28.719	38,05	–18,46	
Viúvo	28.478	27,39	–0,53	20.826	27,59	0,62	
Separado	7.575	7,28	–6,10	6.448	8,54	7,16	
**Escolaridade**							< 0,001
Analfabeto	9.365	10,77	–1,01	7.190	11,02	1,17	
1 a 3 anos	25.243	28,92	5,87	17.315	26,55	–6,78	
4 – 7 anos	23.509	27,04	–1,71	18.079	27,72	1,98	
8 – 11 anos	18.941	21,79	–0,34	14.275	21,89	0,39	
≥ 12 anos	9.982	11,48	–4,94	8.366	12,83	5,71	
**Cor da pele/etnia**							< 0,001
Branca	64.689	61,21	0,45	46.734	60,96	–0,53	
Preta	7.791	7,37	1,79	5.383	7,02	–2,10	
Amarela	950	0,9	–1,98	798	1,04	2,33	
Parda	32.186	30,46	–1,17	23.715	30,93	1,37	
Indígena	60	0,06	0,35	39	0,05	–0,41	

* Teste qui-quadrado.

† Resíduos padronizados do teste qui-quadrado.

Fonte: Datasus. Estatísticas vitais

Os óbitos na faixa de escolaridade superior (> 12 anos) foram menos prevalentes no grupo intra-hospitalar que no grupo extra-hospitalar (11,5% a 12,8%). A cor da pele foi a característica com menor diferença entre os grupos, com discreta redução no número de indivíduos negros no grupo extra-hospitalar (Tabela 2).

Os modelos de regressão binomial negativa e com efeitos fixos obtiveram melhor ajuste em ambos os grupos. Os valores do AIC para cada um dos modelos com efeitos fixos e aleatórios estão descritos na Tabela 3.

**Tabela 3:** Valor do critério de informação de Akaike (AIC) para os modelos de regressão* de Poisson e binomial negativa com os óbitos por infarto agudo do miocárdio ocoridos nas capitais brasileiras de 2007 a 2016, nos grupos intra-hospitalar e extra-hospitalar

	Intra-hospitalar		Extra-hospitalar	
	Poisson	Binomial negativa	Poisson	Binomial negativa
Efeitos fixos	2.344	2.137	3.458	2.339
Efeitos aleatórios	2.778	2.565	3.893	2.777

* Variáveis independentes: residir nas regiões Sul e Sudeste, índice de desenvolvimento humano municipal, expectativa de anos de estudo e índice de Gini.

Para o grupo intra-hospitalar, o modelo de regressão mostrou que o maior IDHM reduziu a incidência de óbitos (IRR = 0,44; IC 95% = 0,33-0,58), enquanto a maior expectativa de anos de estudo associou-se com a elevação da incidência (IRR = 1,09; IC 95% = 1,03-1,15).

O fato de o grupo extra-hospitalar residir nas regiões Sul e Sudeste aumentou a indicência dos óbitos (IRR = 2,84; IC 95% = 1,67-4,85), enquanto a maior expectativa de anos de estudo associou-se com a redução dos óbitos (IRR = 0,86; IC 95% = 0,77-0,97).

O índice de Gini não apresentou diferenças estatisticamente significativas em nenhum dos dois grupos. Os resultados dos modelos de regressão para os dois grupos estão descritos na Tabela 4.

**Tabela 4:** Resultado dos modelos de regressão múltipla binomial negativa com ajuste temporal segundo local de ocorrência dos óbitos por infarto agudo do miocárdio em cada uma das capitais brasileiras de 2007 a 2016. Modelos ponderados pela população de cada capital e analisados com efeitos fixos

	Intra-hospitalar			Extra-hospitalar		
	IRR*	p	IC (95%)	IRR*	p	IC (95%)
Regiões Sul/Sudeste	0,90	0,752	0,49; 1,67	2,84	< 0,001	1,67; 4,85
IDHM†	0,44	< 0,001	0,33; 0,58	1,26	0,347	0,77; 2,07
Expectativa de anos de estudo	1,09	0,004	1,03; 1,15	0,86	0,017	0,77; 0,97
Índice de Gini‡	0,28	0,102	0,60; 1,28	1,02	0,988	0,05; 20,39

* IRR: Incidence Rate Ratio = razão de incidência.

† IDHM: Índice de desenvolvimento humano municipal.

‡ Índice ou coeficiente de Gini: avalia a desigualdade na distribuição de renda. Valores maiores demonstram maior desigualdade.

## Discussão

Os óbitos intra e extra-hospitalares por IAM apresentaram diferenças sociodemográficas e em relação aos índices municipais de desenvolvimento pesquisados neste estudo. A avaliação das capitais brasileiras garante que os óbitos não ocorreram por indisponibilidade de serviços especializados para o tratamento de IAM e caracteriza uma amostra com abrangência nacional, haja vista que nas capitais residem 23,8% da população brasileira.^16^

A prevalência de óbitos por IAM é alta. Estudos anatomopatológicos mostram que de todas as paradas cardíacas extra-hospitalares, o IAM é responsável por quase metade de todas as mortes quando são consideradas todas as idades, proporção que se eleva progressivamente com a idade.^17^ Além disso, a associação de dor precordial com parada cardíaca subsequente mostra uma acurácia próxima a 100% para o diagnóstico de IAM em alguns trabalhos anatomopatológicos.^18^ Na prática clínica, sabemos que a dissecção de aorta, tromboembolismo pulmonar e outras causas agudas também podem cursar com dor precordial e óbito em curto prazo de tempo se mal classificadas, embora sejam bem menos prevalentes que o IAM.^3^^,^^4^

Poucos estudos abordaram especificamente os óbitos extra-hospitalares, justamente pela falta de registros médicos e pela dificuldade de validação de dados. A maior parte dos autores considera o SIM um sistema confiável,^19^^,^^20^ embora haja um maior quantidade de óbitos extra-hospitalares por causas mal definidas, o que pode significar uma menor acurácia do SIM em relação a esses eventos.^21^ Sabe-se também que o SIM não disponibiliza dados abertos se a *causa mortis* foi confirmada por Serviço de Verificação de Óbito (SVO), além de que algumas capitais como Rio de Janeiro, Brasília e Belo Horizonte ainda não haviam implantado um sistema de SVO próprio até o final do ano de 2016.^22^

A literatura mostra uma tendência mundial à redução das taxas de mortalidade por IAM a partir da década de 1960 e desde a década de 1990 no Brasil.^1^^,^^3^ Neste estudo, no entanato, a análise da curva de tendência para o conjunto de todas as capitais mostrou que a mortalidade por IAM intra-hospitalar apresenta-se estável, com discreta tendência à redução, enquanto a mortalidade extra-hospitalar cresceu no período em estudo. A análise detalhada destas tendências podem ser feita mediante o uso de ferramenta analítica específica, o que foge ao objetivo deste trabalho.

As taxas de mortalidade intra-hospitalares são maiores na região Sudeste, em algumas capitais do Nordeste (Natal, João Pessoa e Recife) e em Porto Alegre. Já a mortalidade extra-hospitalar é maior na região Sul, no Rio de Janeiro, em Campo Grande e nas mesmas capitais do Nordeste em que a mortalidade intra-hospitalar é mais alta. Entre todas as capitais, a cidade de Recife se destacou com uma contrastante alta mortalidade em relação às outras capitais do Nordeste, com uma taxa global de óbito inferior apenas ao Rio de Janeiro.

A principal hipótese dos estudos que visam explicar uma mortalidade extra-hospitalar mais alta é o tempo maior entre o início dos sintomas e a chegada ao hospital. Uma revisão sistemática, publicada em 2010, abordou 42 estudos e mostrou que pacientes do sexo feminino e idosos demorariam mais para receber tratamento hospitalar.^23^ Paradoxalmente a estes dados, encontramos em nosso estudo evidências de que a mortalidade extra-hospitalar foi comparativamente maior entre indivíduos do sexo masculino e em pacientes com mais de 80 anos. Mais de 70% dos óbitos ocorreram em idosos (> 60 anos) e os pacientes do sexo masculino apresentaram maior mortalidade por IAM nos dois grupos.

Outros estudos mostraram que pacientes casados demorariam menos tempo até chegar ao hospital.^24^^,^^25^ Nossos resultados mostram que a mortalidade extra-hospitalar foi menor nos casados, provavelmente por disponibilizarem de companheiro(a) para levá-los até uma instituição hospitalar.

A mortalidade extra-hospitalar foi ligeiramente maior em pacientes com nível de escolaridade superior. Embora pessoas com maior escolaridade apresentem uma taxa de sobrevivência maior após um episódio de IAM,^26^^,^^27^ este fator pode não influenciar de sobremaneira o episódio agudo, pois o atendimento inicial não especializado e mesmo a automedicação podem retardar o tratamento apropriado.^28^^,^^29^

Um maior IDHM associou-se com a redução da mortalidade intra-hospitalar (IRR = 0,44; IC 95% = 0,33-0,58), sem efeito sobre a mortalidade extra-hospitalar. Provavelmente, há maior disponibilidade e qualidade de recursos terapêuticos nas cidades com maior IDHM. Estudos comparativos mostram que países com melhor Produto Interno Bruto (PIB) dispunham de mais recursos terapêuticos e apresentavam menor mortalidade por IAM.^30^ Da mesma forma, análises espaciais realizadas em cidades brasileiras mostraram o aumento da mortalidade por IAM em bairros mais pobres.^7^^,^^31^^,^^32^ Uma análise espacial no município do Rio de Janeiro mostrou que um IDH menor e calculado por bairros foi um importante fator de risco para os óbitos por doenças cerebrovasculares, as quais compartilham fisiopatologia e fatores de risco semelhantes ao IAM.^8^

Residir nas regiões Sul e Sudeste aumentou a incidência dos óbitos extra-hospitalares (IRR = 2,84; IC 95% =1,67-4,85). Observou-se também que em todas as capitais da região Sul e no Rio de Janeiro os óbitos extra-hospitalares são mais prevalentes que os intra-hospitalares. Este achado pode ter várias hipóteses explicativas. Uma delas é que os serviços de saúde nestas regiões se encontram melhor estruturados, o que explicaria em parte a redução de óbitos intra-hospitalares com um maior IDHM. Como a taxa de mortalidade intra-hospitalar é menor, predominaram os óbitos de pacientes que não conseguiram receber atendimento em tempo hábil.

Outra hipótese é que algumas destas capitais apresentam uma população idosa maior, mais suscetíveis a IAM e com menor capacidade de locomoção, além do fato de serem maiores e mais populosas, o que torna o acesso aos serviços de saúde e a agilidade no transporte dos enfermos um grande desafio logístico.^21^^,^^33^ Além disso, o estilo de vida insalubre, a maior taxa de tabagismo, o maior estresse cotidiano, a dieta inadequada e uma maior taxa de inatividade física, fatores estes que se associam com a urbanização excessiva, podem aumentar o risco de IAM,^34^^–^^36^ o que justificaria também as maiores taxas de mortalidade nestas cidades.

A expectativa de anos de estudo mostrou resultados opostos em relação aos grupos intra e extra-hospitalar. As capitais com maior expectativa de anos de estudo apresentaram mais óbitos intra-hospitalares (IRR = 1,09; IC 95% = 1,03-1,15) e menos óbitos extra-hospitalares (IRR = 0,86; IC 95%=0,77-0,97). O estudo AFIRMAR abordou os fatores de risco para IAM no Brasil e mostrou que o nível educacional superior correlacionou-se com um menor risco de IAM (OR = 0,68 e p = 0,0239) apenas quando a renda do paciente era mais elevada.^37^ Ainda que em nossa pesquisa tivessem sido registrados mais óbitos extra-hospitalares nos níveis mais altos de escolaridade, uma cidade com expectativa de anos de estudo maior, provavelmente, tem maior acesso à informação, melhor conhecimento de sinais e sintomas e, consequentemente, um deslocamento dos óbitos do extra-hospitalar para o intra-hospitalar.

São pontos fortes deste estudo as novas contribuições para o entendimento da dinâmica dos óbitos por IAM, principalmente dos extra-hospitalares, dos quais pouco se sabe. A escolha das capitais como fator de amostragem garante um representante para cada unidade federativa brasileira e a cobertura de 23,8% da população do Brasil.

A utilização de modelos de regressão binomial negativa com ajuste temporal e ponderadas pelo tamanho da população possuem a vantagem de possibilitar que cada capital tenha seu próprio intercepto e que sirva como seu próprio controle, permitindo o ajuste para variáveis não mensuradas e que não variam ao longo do tempo, além de possibilitar a modelagem direta do número de eventos ao invés das taxas, já que estas podem sofrer variações em função das mudanças no numerador ou no denominador.

As limitações deste estudo incluem o uso de uma abordagem ecológica e conveniente para analisar uma série temporal, além da menor qualidade dos dados referentes aos óbitos extra-hospitalares. Outra limitação foi o uso de índices municipais de desenvolvimento obtidos através do censo demográfico que, embora sejam uma alternativa, não consideram as variações e flutuações de índice ocorridas no intervalo entre as coletas.

## Conclusão

Este estudo trouxe novas informações sobre os óbitos por IAM nas capitais. Os óbitos intra e extra-hospitalares apresentam diferenças em relação às tendências temporais, às características sociodemográficas, ao IDHM, à expectativa de anos de estudo e quanto ao fato de residir nas regiões Sul e Sudeste.

Paradoxalmente ao reportado na literatura em relação a mortalidade global por IAM, a mortalidade extra-hospitalar está aumentando nas capitais brasileiras. Quando comparada ao grupo intra-hospitalar, a mortalidade extra-hospitalar acomete mais homens, pessooas com mais de 80 anos e não casados. A educação se mostrou um fator de deslocamento da mortalidade de um grupo para o outro. Residir nas regiões Sul e Sudeste mostrou associação com maior incidência de óbitos extra-hospitalares, enquanto que um maior IDHM está ligado com a menor incidência de óbitos intra-hospitalares, sem efeito estatisticamente significativo sobre os óbitos extra-hospitalares. Estudos posteriores são necessários para averiguar se estas diferenças também ocorrem no interior do país, onde as condições na maioria das cidades para tratamento do IAM são mais precárias. Os dados apresentados neste estudo ajudam a conhecer melhor a realidade e as tendências da mortalidade nas capitais brasileiras, podendo políticas públicas para reduzi-la pela mais prevalente causa de óbito.
