# Supplementary material for: Óbitos Intra e Extra-Hospitalares por Infarto Agudo do Miocárdio nas Capitais Brasileiras
Source: Arq Bras Cardiol. 2021 Aug 9;117(2):319–26. [Article in Portuguese] doi: 10.36660/abc.20200043 (PMC8395787; doi:10.36660/abc.20200043)
Supplement: Translation

# Translation: In- and Out-of-Hospital Deaths by Acute Myocardial Infarction in Brazilian State Capitals

**Authors:** Sterffeson Lamare Lucena de Abreu, Joana D’Arc Matos França de Abreu, Maria dos Remédios Freitas Carvalho Branco, Alcione Miranda dos Santos

**Affiliations:** 1 São LuísMA Brazil Hospital Universitário da Universidade Federal do Maranhão, São Luís, MA - Brazil; 2 São LuísMA Brazil Universidade Federal do Maranhão - Pós Graduação em Saúde Coletiva, São Luís, MA - Brazil

**Keywords:** Myocardial Infarction, Out-of-Hospital, Epidemiology, Deaths, Demographic Indicators, Social Indicators, Mortality, Death, Sudden Cardiac

## Abstract

**Background:**

Acute myocardial infarction (AMI) is the main cause of death in Brazil and the world. Approximately half of these deaths occur outside the hospital.

**Objectives:**

To analyze the distribution, temporal evolution, and sociodemographic characteristics (SDC) of in- and out-of-hospital deaths by AMI in Brazilian state capitals and their relationship with municipal development indicators (MDI).

**Methods:**

This is an ecological study of the number of deaths due to AMI reported annually by the 27 Brazilian state capitals from 2007 to 2016; these were divided into 2 groups: in-hospital (H) and out-of-hospital (OH). We evaluated the temporal evolution of mortality rates in each group and differences in SDC. Negative binomial regression models were used to compare the temporal evolution of the number of deaths in each group with the following variables: residing in the South/Southeast regions (S/SE), municipal human development index (MHDI), Gini coefficient, and expected years of schooling (EYS). We considered p-values<0.05 as statisticallysignificant.

**Results:**

The OH mortality rate increased with time for all state capitals. All studied SDC were different between groups (p<0.001). In the OH group, most deaths were of men and patients aged 80 years or older and not married. S/SE increased the incidence of OH deaths (incidence rate ratio [IRR]=2.84; 95% confidence interval [CI]=1.67–4.85), while higher EYS reduced it (IRR=0.86; 95% CI=0.77–0.97). In the H group, higher MHDI reduced the incidence of deaths (IRR=0.44; 95% CI=0.33–0.58), while higher EYS increased it (IRR=1.09; 95% CI=1.03–1.15).

**Conclusions:**

In- and out-of-hospital deaths due to AMI in Brazilian state capitals were influenced by MDI, presented sociodemographic differences and a progressive increase in out-of-hospital occurrences.

## Introduction

Acute myocardial infarction (AMI) is the main individual cause of death in Brazil and the world.^1^^,^^2^ It has a mean mortality of 30% when untreated and of less than 6% when appropriate treatment is administered in time.^3^ Half of these deaths occur within the first 2 hours of symptom onset and 80% happen in the first 24 hours, resulting in many deaths before any hospital care.^4^

Appropriate treatment of high-risk AMI is costly, and its availability is concentrated in large urban areas, mainly in state capitals; this is especially true in the North, Northeast, and Central-West regions of Brazil.^5^ Although epidemiological studies have shown that mortality due to AMI is slowly decreasing worldwide, this reduction is smaller in countries with lower Gross Domestic Products (GDPs), lower social classes, and socioeconomically disadvantaged regions.^6^^–^^8^

Few studies have been published on out-of-hospital deaths due to AMI. Most of them consider general mortality without distinguishing between in-hospital and out-of-hospital deaths. Clinical studies on risk factors have been performed with patients who received hospital care. It is unknown whether deaths occurring out of the hospital environment presented sociodemographic differences in comparison with those who happened within a hospital, and the association of local and environmental factors with out-of-hospital mortality is still not well defined.^9^^,^^10^

The aim of this study was to temporally analyze in- and out-of-hospital deaths due to AMI in Brazilian state capitals, identifying sociodemographic differences and considering municipal development indices. We chose to assess only the state capitals because all of them currently provide advanced treatment of AMI.^11^

## Method

This is an ecological study of deaths due to AMI occurred in the 27 Brazilian state capitals between 2007 and 2016. Data on deaths per state capital (in- or out-of-hospital occurrence, sex, age group, schooling, marital status, and skin color) were obtained from the Mortality Information System (SIM), an online platform created by the informatics department of the Unified Health System (DATASUS) for regular retrieval of mortality data in Brazil. Deaths were divided into 2 groups according to the place of occurrence: in-hospital or out-of-hospital.

For selecting deaths due to AMI in the SIM, we considered entries that had AMI as the primary cause of death (International Classification of Diseases [ICD]-10: I21). Deaths with unknown place of occurrence were not included in this study.

In- and out-of-hospital mortality rates were obtained by calculating the rate between deaths due to AMI and the population of each state capital (per 100 000 inhabitants). These rates are presented as means, standard deviations (SDs), and minimum and maximum values.

To assess the temporal evolution of mortality rates in both groups, we calculated annual in- and out-of-hospital mortality rates for all Brazilian state capitals. The population was corrected by linear interpolation and extrapolation using data from demographic census of 2000, 2010, and the 2017 projection made by the Brazilian Institute of Geography and Statistics (IBGE). Rates were presented as deaths per 100 000 inhabitants and expressed as a line graph.

The Atlas Brasil platform of the United Nations Development Program (PNUD) was used for obtaining independent variables (municipal human development index [MHDI], Gini coefficient, and expected years of schooling), as well as information on the population of each state capital.^12^

### Statistical analysis

For comparing the number of deaths in both groups according to sociodemographic characteristics (sex, age group, schooling, marital status, and skin color), we used the chi-squared test. Sociodemographic characteristics were presented as absolute and relative frequencies. To demonstrate the impact of each characteristic, we calculated the standardized residuals of chi-squared tests, which are presented as Z in Table 2. Considering a significance level of 5%, Z-values > +1.96 or < −1.96 were statistically significant and the plus and minus signs showed the direction of differences between groups.

To verify which independent variables were associated with the number of deaths in both groups, we used the panel data methodology, in which information from various sampling units (each state capital) was assessed through time, that is, observations were considered in 2 dimensions: the sampling unit and time.^13^ Therefore, we used Poisson and negative binomial regression models with temporal adjustment and weighted by the population of each capital for each of the groups. Weighting was performed according to the population of each capital so that each sampling unit had the same weight when evaluating associations.

The models were tested with fixed and random effects. Those with fixed effects led to each capital having its own intercept, serving as its own control, which allowed the adjustment for unmeasured variables that did not change with time (such as census data, which are updated every 10 years).^13^

For choosing the model with the best fit, we considered the Akaike Information Criterion (AIC).^14^ The lower the AIC, the better the fit. We also estimated the incidence rate ratio (IRR) and its respective confidence interval, considering as reference a 95% confidence interval (95% CI). Statistical analysis was performed using Stata software, version 14.0. This study only used data available in the public domain, thus not requiring assessment by a research and ethics committee as it does not fit the terms of Resolution 466, of December 2012.^15^

## Results

Between 2007 and 2016, 189 634 deaths due to AMI were reported in Brazilian state capitals; 41.7% of them were out-of-hospital deaths. The mean mortality rate per 100 000 inhabitants in state capitals was 25.2 ± 1.3 for in-hospital deaths and 18 ± 1.2 for out-of-hospital deaths. The temporal evolution of the annual rate for all capitals in both groups is demonstrated in Figure 1.

**Figure 1:** Temporal evolution of in- and out-of-hospital mortality rates due to acute myocardial infarction (AMI) per 100 000 inhabitants. Brazilian state capitals, 2007–2016. IBGE: Brazilian Institute of Geography and Statistics.

Source: DATASUS. Vital statistics.

The highest and lowest mean death rates were reported in Recife (43.2%) and Palmas (8.7%), respectively, for the in-hospital group, and in Rio de Janeiro (33.8%) and Macapá (4.7%) for the out-of-hospital group (Table 1). In many state capitals, out-of-hospital deaths were more prevalent than in-hospital deaths: Palmas, São Luís, Rio de Janeiro, Curitiba, Florianópolis, Porto Alegre, and Campo Grande.

**Table 1:** Mortality rates due to acute myocardial infarction in Brazilian state capitals from 2007 to 2016 (deaths/100 000 inhabitants). Mean, standard deviation (SD), and minimum (Min) and maximum (Max) recorded values

	In-hospital (H)			Out-of-hospital (OH)			% OH
	Mean	SD	Min – Max	Mean	SD	Min – Max	Mean
Porto Velho	13.12	2.38	10.15 – 18.53	12.14	5.34	6.28 – 22.40	48.06%
Rio Branco	14.17	3.40	10.42 – 19.33	10.04	3.52	5.95 – 14.88	41.47%
Manaus	14.35	1.86	11.71 – 17.32	4.88	2.07	2.22 – 9.10	25.38%
Boa Vista	12.49	2.83	7.39 – 16.18	9.35	1.71	5.63 – 11.61	42.81%
Belém	18.51	3.07	14.99 – 23.47	17.53	3.67	12.49 – 22.39	48.64%
Macapá	10.44	3.26	5.52 – 16.83	4.74	3.56	1.00 – 11.55	31.23%
Palmas	8.68	2.26	4.82 – 11.65	13.43	5.11	5.69 – 22.57	60.74%
São Luís	18.66	2.15	15.96 – 21.93	20.17	4.45	13.30 – 26.90	51.94%
Teresina	21.40	1.85	18.08 – 25.05	13.84	2.96	10.93 – 20.90	39.27%
Fortaleza	16.88	1.33	14.35 – 18.19	6.38	1.86	3.71 – 9.42	27.43%
Natal	23.46	2.68	21.52 – 30.11	23.40	5.75	16.32 – 31.85	49.94%
João Pessoa	25.17	1.70	21.84 – 27.37	21.76	3.01	17.97 – 25.98	46.37%
Recife	43.16	5.54	36.96 – 51.37	21.23	2.21	15.95 – 23.61	32.97%
Maceió	17.77	1.91	14.69 – 20.05	14.20	2.94	10.29 – 18.80	44.42%
Aracajú	17.02	1.82	14.53 – 20.37	11.82	2.80	8.58 – 18.38	40.98%
Salvador	16.19	1.48	13.04 – 17.98	9.47	1.72	6.65 – 13.49	36.91%
Belo Horizonte	15.00	1.48	13.01 – 17.56	9.11	0.60	7.75 – 9.94	37.79%
Vitória	21.70	4.37	15.56 – 27.65	18.34	1.40	16.04 – 19.83	45.80%
Rio de Janeiro	32.68	2.55	29.35 – 38.17	33.75	2.61	27.93 – 36.72	50.81%
São Paulo	36.41	2.07	33.62 – 39.72	17.84	1.73	15.84 – 20.66	32.88%
Curitiba	16.87	1.71	14.56 – 18.84	23.42	1.84	20.49 – 25.86	58.13%
Florianópolis	16.55	2.34	12.58 – 19.84	16.95	4.00	10.92 – 24.22	50.60%
Porto Alegre	23.22	1.65	21.07 – 26.90	30.46	3.18	25.33 – 34.84	56.74%
Campo Grande	18.00	1.70	16.12 – 21.48	33.30	10.59	22.75 – 56.81	64.91%
Cuiabá	20.42	1.78	18.62 – 24.13	15.49	4.72	10.52 – 23.59	43.14%
Goiânia	17.57	2.55	13.44 – 21.89	13.43	3.21	9.52 – 19.34	43.32%
Brasília	15.86	1.19	14.36 – 18.23	7.55	2.46	4.20 – 12.10	32.25%

Source: DATASUS. Vital statistics.

Both groups were statistically different for all the studied sociodemographic characteristics (Table 2). When comparing groups, deaths of male patients were more frequent in the out-of-hospital group (57.4% vs 55.5%). Regarding age groups, the out-of-hospital group presented more deaths of individuals aged over 80 years (29.7% vs 26.3%). Married patients had fewer out-of-hospital deaths (38% vs 46%) (Table 2).

**Table 2:** Sociodemographic distribution of in- and out-of-hospital deaths due to acute myocardial infarction. Brazilian state capitals, 2007–2016

	In-hospital			Out-of-hospital			p-value *
	n (110 549)	%	Z†	n (79 085)	%	Z†	
**Sex**							< 0.001
Male	61304	55.45	–3.58	45389	57.39	4.24	
Female	49245	44.55	4.06	33696	42.61	–4.81	
**Age Group**							< 0.001
< 1 year	50	0.05	3.43	3	0	–4.06	
1 – 4 years	3	0	0.95	0	0	–1.12	
5 – 9 years	2	0	0.19	1	0	–0.22	
10 – 14 years	14	0.01	–0.15	11	0.01	0.18	
15 – 19 years	207	0.19	3.73	67	0.08	–4.42	
20 – 29 years	685	0.62	0.97	447	0.57	–1.14	
30 – 39 years	1877	1.7	–6.02	1821	2.31	7.12	
40 – 49 years	6991	6.33	–6.10	5904	7.47	7.22	
50 – 59 years	17 580	15.91	–0.98	12 788	16.19	1.16	
60 – 69 years	25 204	22.81	4.73	16 745	21.20	–5.60	
70 – 79 years	28 847	26.10	10.22	17 729	22.45	–12.09	
≥ 80 years	29 052	26.29	–9.02	23 471	29.72	10.67	
**Marital status**							< 0.001
Single	20 517	19.73	–17.49	19 489	25.82	20.53	
Married	47 417	45.60	15.72	28 719	38.05	–18.46	
Widowed	28 478	27.39	–0.53	20 826	27.59	0.62	
Separated	7575	7.28	–6.10	6448	8.54	7.16	
**Schooling**							< 0.001
Illiterate	9365	10.77	–1.01	7190	11.02	1.17	
1 – 3 years	25 243	28.92	5.87	17 315	26.55	–6.78	
4 – 7 years	23 509	27.04	–1.71	18 079	27.72	1.98	
8 – 11 years	18 941	21.79	–0.34	14 275	21.89	0.39	
≥ 12 years	9982	11.48	–4.94	8366	12.83	5.71	
**Skin color/ethnicity**							< 0.001
White	64 689	61.21	0.45	46 734	60.96	–0.53	
Black	7791	7.37	1.79	5383	7.02	–2.10	
Yellow	950	0.9	–1.98	798	1.04	2.33	
Brown	32 186	30.46	–1.17	23 715	30.93	1.37	
Indigenous	60	0.06	0.35	39	0.05	–0.41	

* Chi-squared test.

† Standardized residuals of the chi-squared test.

Source: DATASUS. Vital statistics.

Deaths of people with higher levels of schooling (> 12 years) were less prevalent in the in-hospital group than in the out-of-hospital group (11.5% vs 12.8%). Skin color was the characteristic with the smallest difference between groups: a discrete reduction in black individuals was observed in the out-of-hospital group (Table 2).

The negative binomial regression models with fixed effects provided better fit for both groups. AIC values for each model with fixed and random effects are described in Table 3.

**Table 3:** Akaike Information Criterion (AIC) values for the Poisson and negative binomial regression models* regarding deaths due to acute myocardial infarction occurred in Brazilian state capitals from 2007 to 2016 in the in- and out-of-hospital groups

	In-hospital		Out-of-hospital	
	Poisson	Negative binomial	Poisson	Negative binomial
Fixed effects	2344	2137	3458	2339
Random effects	2778	2565	3893	2777

* Independent variables: residing in the South and Southeast regions, municipal human development index, expected years of schooling, and Gini coefficient.

For the in-hospital group, the regression model showed that a higher MHDI reduced the incidence of deaths (IRR = 0.44; 95% CI = 0.33–0.58), while higher expected years of schooling were associated with higher incidence (IRR = 1.09; 95% CI = 1.03–1.15).

For the out-of-hospital group, residing in the South and Southeast regions increased the incidence of deaths (IRR = 2.84; 95% CI = 1.67–4.85), while higher expected years of schooling were associated with a reduction in deaths (IRR = 0.86; 95% CI = 0.77–0.97).

The Gini coefficient did not present statistically significant differences between groups. The results of regression models for both groups are presented in Table 4.

**Table 4:** Results of negative binomial multiple regression models with temporal adjustment according to the place of occurrence of deaths due to acute myocardial infarction in each of the Brazilian state capitals from 2007 to 2016. Models were weighted by the population of each capital and analyzed with fixed effects.

	In-hospital			Out-of-hospital		
	IRR*	p	CI (95%)	IRR*	p	CI (95%)
South/Southeast regions	0.90	0.752	0.49; 1.67	2.84	< 0.001	1.67; 4.85
MHDI†	0.44	< 0.001	0.33; 0.58	1.26	0.347	0.77; 2.07
Expected years of schooling	1.09	0.004	1.03; 1.15	0.86	0.017	0.77; 0.97
Gini coefficient‡	0.28	0.102	0.60; 1.28	1.02	0.988	0.05; 20.39

* IRR: incidence rate ratio.

† MHDI: municipal human development index.

‡ Gini coefficient: assesses inequality in income distribution. Higher values demonstrate higher inequality.

## Discussion

In- and out-of-hospital deaths due to AMI presented differences regarding the sociodemographic characteristics and municipal development indices considered in this study. The assessment of Brazilian state capitals guaranteed that deaths did not happen due to unavailability of specialized care and characterized a nation-wide coverage of the sample, since state capitals account for 23.8% of the Brazilian population.^16^

The prevalence of deaths due to AMI is high. Anatomopathological studies show that, of all out-of-hospital cardiac arrests, AMI is responsible for almost half the deaths when considering all ages; this proportion increases progressively with age.^17^ In addition, the association of precordial pain with subsequent cardiac arrest shows near 100% accuracy for AMI diagnosis in some anatomopathological studies.^18^ In clinical practice, it is known that aortic dissection, pulmonary thromboembolism, and other acute or potentially acute causes can also progress with precordial pain and death in a short period of time and could be misclassified, but these are much less prevalent than AMI.^3^^,^^4^

Few studies have specifically approached out-of-hospital deaths precisely due to the lack of medical records and difficulties in validating data. Most authors consider the SIM as a reliable system^19^^,^^20^ even though out-of-hospital deaths are more frequently reported as having ill-defined causes, which could represent a lower accuracy of the SIM regarding these events.^21^ It is also known that SIM does not provide, as open data, whether the causa mortis was confirmed by the Death Verification Service (SVO), and some state capitals such as Rio de Janeiro, Brasília, and Belo Horizonte had not implemented their own SVO until late 2016.^22^

The literature shows a trend of reduction in mortality rates due to AMI since the 1960s worldwide and since the 1990s in Brazil.^1^^,^^3^ However, in this study, analysis of the trend curve showed that in-hospital mortality due to AMI is stable, with a slight trend of reduction, while out-of-hospital mortality increased in the studied period. A detailed analysis of these trends can be performed with specific analytic tools, which is not within the scope of this study.

In-hospital mortality rates are higher in the Southeast region, in some capitals of the Northeast region (Natal, João Pessoa, and Recife), and in Porto Alegre. On the other hand, out-of-hospital mortality is higher in the South region, in Rio de Janeiro, Campo Grande, and the same northeastern capitals where in-hospital mortality is higher. Recife stood out with a notably high mortality when compared to other northeastern capitals, with a global death rate that was only lower than that of Rio de Janeiro among all capitals.

The main hypothesis of studies explaining higher out-of-hospital mortality is a longer time between symptom onset and arrival at the hospital. A systematic review published in 2010 considered 42 studies and observed that women and older patients took longer to receive hospital treatment.^23^ Paradoxically, in our study we observed that out-of-hospital mortality was higher in men and in patients aged over 80 years. More than 70% of deaths occurred among older people (> 60 years), and men presented higher mortality due to AMI in both groups.

Other studies observed that married patients took less time to arrive at the hospital.^24^^,^^25^ Our results indicated that out-of-hospital mortality was lower in married patients, probably because these had a partner that could help them access hospital care.

Out-of-hospital mortality was slightly higher in patients with higher levels of schooling. Although people with higher levels of schooling have higher survival rates after an AMI,^26^^,^^27^ this may not significantly affect the acute episode, since initial care by a non-specialist and even self-medication may delay proper care.^28^^,^^29^

A higher MHDI is associated with a reduction in in-hospital mortality (IRR = 0.44; 95% CI = 0.33–0.58), with no effect on out-of-hospital mortality. Cities with higher MHDI probably have greater availability and quality of therapeutic resources. Studies that compared countries showed that countries with higher GDP had higher availability of therapeutic resources and lower mortality by AMI.^30^ Similarly, spatial analyses performed in Brazilian cities showed an increase in mortality by AMI in poorer neighborhoods.^7^^,^^31^^,^^32^ A spatial analysis performed in Rio de Janeiro observed that lower HDI, calculated for each neighborhood, was an important risk factor for deaths due to cerebrovascular diseases, which share their physiopathology and risk factors with AMI.^8^

Residing in the South and Southeast regions increased the incidence of out-of-hospital deaths (IRR = 2.84; 95% CI = 1.67–4.85). We observed that, in all capitals of the South region and in Rio de Janeiro, out-of-hospital deaths were more prevalent than in-hospital deaths. This finding can be explained by various hypotheses. One of them is that health care services in these regions are better equipped, which could partially explain the reduction in in-hospital deaths in cities with a higher MHDI. Since the in-hospital mortality rate is lower, deaths of patients that could not receive timely care prevailed.

Another hypothesis is that some of these capitals present a larger older population, more susceptible to AMI and with lower locomotion abilities, in addition to the fact that these cities are larger and more densely populated, which could represent a great logistical challenge regarding the access to health care services and fast transportation of sick patients.^21^^,^^33^ Moreover, the unhealthy lifestyle, inadequate diet, and higher smoking rate, daily stress, and physical inactivity rate associated with excessive urbanization may increase the risk of AMI,^34^^–^^36^ which could also justify higher mortality rates due to AMI in these cities.

Expected years of schooling showed opposite results between in- and out-of-hospital groups. Capitals with higher expected years of schooling presented more in-hospital deaths (IRR = 1.09; 95% CI = 1.03–1.15) and less out-of-hospital deaths (IRR = 0.86; 95% CI = 0.77–0.97). The AFIRMAR study considered risk factors for AMI in Brazil and showed that higher schooling was correlated with a lower risk of AMI (odds ratio [OR] = 0.68; p = 0.0239) only when the patient's income was higher.^37^ Although in our study there were more out-of-hospital deaths among patients with higher levels of schooling, the inhabitants of a city with higher expected years of schooling probably have better access to information, with better knowledge on signs and symptoms, resulting in a change from out-of-hospital deaths to in-hospital deaths.

Strengths of this study include new contributions to understanding the dynamics of deaths by AMI, especially the out-of-hospital ones, which are little known. The choice of state capitals as sample guarantees the representation of every Brazilian federative unit and coverage of 23.8% of the Brazilian population.

The use of negative binomial regression models with temporal adjustment and weighted by population size has the advantage of letting each capital have its own intercept, serving as its own control, which allows the adjustment for unmeasured variables that do not change with time, in addition to the possibility of directly modelling the number of events instead of rates, which can suffer variations according to changes in numerators and denominators.

Limitations of this study include the use of an ecological and convenience approach for analyzing a time series, in addition to the lower quality of data regarding out-of-hospital deaths. Another limitation involved the use of municipal development indices obtained by the demographic census that, although consist of an alternative for estimation, do not consider the variations and fluctuations that occurred during the interval between data collections.

## Conclusion

This study brought new information regarding deaths by AMI in state capitals. In- and out-of-hospital deaths presented differences in temporal trends, sociodemographic characteristics, MHDI, expected years of schooling, and whether patients resided in the South and Southeast regions.

As opposed to what is reported by the literature regarding global mortality by AMI, out-of-hospital mortality is increasing in Brazilian capitals. In comparison with the in-hospital group, out-of-hospital mortality affected more men, people older than 80 years, and unmarried people. Schooling was a factor that converted out-of-hospital mortality into in-hospital mortality. Residing in the South and Southeast regions was associated with a higher incidence of out-of-hospital deaths, while higher MHDI was associated with a lower incidence of in-hospital deaths with no statistically significant effect on out-of-hospital deaths. Further studies are necessary to verity if these differences also happen in other cities, where conditions for AMI treatment are generally more precarious.

Data presented in this study have helped us better understand the reality and trends of mortality in Brazilian state capitals and may contribute to guiding public policies for reducing mortality due to the most prevalent cause of death.
